# Clinical and Radiographic Evaluation of Intrabony Periodontal Defects Treated with Hyaluronic Acid or Enamel Matrix Proteins: A 6-Month Prospective Study

**DOI:** 10.3290/j.ohpd.b5569745

**Published:** 2024-07-12

**Authors:** Octavia-Carolina Vela, Marius Boariu, Darian Rusu, Vincenzo Iorio-Siciliano, Anton Sculean, Stefan-Ioan Stratul

**Affiliations:** a Assistant Professor, Department of Periodontology, Faculty of Dental Medicine, Anton Sculean Research Center for Periodontal and Peri-implant Diseases, Victor Babes University of Medicine and Pharmacy, Timisoara, Romania. Conception, design, analysis, interpretation, treated patients, drafted and critically revised the manuscript.; b Associate Professor Department of Endodontics, Faculty of Dental Medicine, TADERP Research Center, Victor Babes University of Medicine and Pharmacy, Timisoara, Romania. Study design, analysis, interpretation, drafted and critically revised the manuscript.; c Professor, Department of Periodontology, Faculty of Dental Medicine, Anton Sculean Research Center for Periodontal and Peri-implant Diseases, Victor Babes University of Medicine and Pharmacy, Timisoara, Romania. Data acquisition, analysis, and interpretation.; d Associate Professor Department of Periodontology, School of Dental Medicine, University of Naples Federico II, Naples, Italy. Data interpretation, critically revised the manuscript.; e Professor, Department of Periodontology, University of Bern, Bern, Switzerland. Data interpretation, critically revised the manuscript.; f Professor, Department of Periodontology, Faculty of Dental Medicine, Anton Sculean Research Center for Periodontal and Peri-implant Diseases, Victor Babes University of Medicine and Pharmacy, Timisoara, Romania. Conception, design, drafted and critically revised the manuscript.; All authors gave their final approval and agreed to be accountable for all aspects of the work.

**Keywords:** cross-linked hyaluronic acid, enamel matrix derivative, intrabony defects, periodontal pocket, periodontal regeneration

## Abstract

**Purpose:**

To compare the regenerative clinical and radiographic effects of cross-linked hyaluronic acid (xHyA) with enamel matrix proteins (EMD) at six months after regenerative treatment of periodontal intrabony defects.

**Materials and Methods:**

Sixty patients presenting one intrabony defect each were randomly assigned into control (EMD) and test (xHyA) groups. Clinical attachment level (CAL) gain was the primary outcome, while pocket probing depth (PPD), gingival recession (REC), bleeding on probing (BOP), full-mouth plaque score (FMPS), full-mouth bleeding score (FMBS), and radiographic parameters such as defect depth (BC-BD), and defect width (DW) were considered secondary outcome variables. Parameters were recorded at baseline and after 6 months.

**Results:**

At the 6-month follow-up, 54 patients were available for statistical analysis. In the control and test groups, the mean CAL gain was statistically significant in the intragroup comparison (p < 0.001). 48.1% of test sites showed a CAL gain ≤ 2 mm compared with 33.3% of control sites. The mean PPD reduction was statistically significant in the intragroup comparison in both groups (p < 0.001). The mean REC increase was similar in the two groups: 1.04 ± 1.29 mm vs 1.11 ± 1.22 mm (test vs control). The mean BC-BD, DW, FMPS, FMBS, and BOP changed statistically significantly only in the intragroup comparison, not in the intergroup comparison.

**Conclusion:**

Both treatments, EMD and xHyA, produced similar statistically significant clinical and radiographical improvements after six months when compared with baseline.

Periodontal disease is an infectious inflammatory condition that can lead to the destruction of the periodontal ligament and loss of alveolar bone support, ultimately resulting in tooth loss if left untreated.^64^^,^^80^ Through its evolution, periodontitis can result in intrabony defects, described as osseous defects with a base located apical to the interdental alveolar crest and enclosed by one, two, or three bony walls or a mixture of them.^42^ Although the periodontium has a strong regenerative potential, if left untreated, these defects represent a risk factor for disease progression that ultimately ends with tooth loss.^65^^,^^91^ If the endpoints of steps 1 and 2 of periodontal therapy are not met, surgical intervention becomes the treatment of choice for deep intrabony defects.^15^^,^^72^^,^^79^ The formation of root cementum, periodontal ligament, and alveolar bone histologically characterises periodontal regeneration.^84^ Better results in terms of clinical, radiographic, and patient-reported outcomes have been observed when regenerative procedures, such as the use of specific bone replacement materials, barrier membranes, enamel matrix derivative (EMD), recombinant platelet-derived growth factor (rhPDGF), or various combinations thereof, are used compared to open flap debridement (OFD) alone.^62^^,^^84^^,^^98^

Hyaluronic acid (HA) is a linear polysaccharide that can be found in the extracellular matrix of several tissues and organs in the body, such as connective tissue, synovial fluid, embryonic mesenchyme, vitreous humor, and skin.^52^ It is also a crucial component found in both the soft periodontal tissues, e.g., gingiva and periodontal ligament, as well as in hard tissues, such as alveolar bone and cementum.^16^

Having a molecular weight ranging from 4000 to 20,000,000 Da, HA is a non-sulfated glycosaminoglycan that occurs naturally. Its structure comprises polyanionic disaccharide units of glucuronic acid and N-acetyl glucosamine, linked by alternating beta-1,3 and beta-1,4 bonds.^32^ Hydrogen bonding occurs between adjacent carboxyl and N-acetyl groups in hyaluronic acid when it is added to an aqueous solution, making it the most hygroscopic molecule in nature. This property helps HA maintain conformational stiffness and retain water. Hyaluronic acid also exhibits important viscoelastic qualities that decrease the infiltration of viruses and bacteria into the tissues.^92^

The molecule plays various structural and physiological roles in these tissues and is the key component in the various stages of the wound-healing process, e.g., inflammation, granulation tissue formation, epithelium formation, and tissue remodeling, in both mineralised and non-mineralised tissues.^1^ The high-molecular-weight hyaluronic acid produced by hyaluronan synthase enzymes in periodontal tissues can regulate the inflammatory response. In chronically inflamed tissues, for instance, gingival tissue inflammation or after implant or sinus-lift surgery, HA undergoes significant degradation into lower molecular weight molecules.^4^^,^^33^ It is plausible to hypothesise that HA performs similar functions in restoring both mineralised and nonmineralised periodontal tissues, given its multifunctional role in wound healing and the similar biological principles underlying gingival and bone regeneration.^10^^,^^27^

Since there have been no reported contraindications or drug interactions with HA to date, multiple HA-based biomaterials have been developed for the treatment of various conditions in different medical fields such as dermatology, ophthalmology and orthopedics^30^^,^^40^^,^^55^^,^^101^ as well as dentistry in the treatment of gingivitis,^37^^,^^46^ non-surgical periodontal therapy^19^^,^^20^ management of furcation involvements,^39^ alveolar ridge preservation,^2^^,^^31^ root coverage procedures,^26^^,^^44^^,^^67^ and mucositis and peri-implantitis management.^47^^,^^48^

The primary objective of periodontal therapy is to regenerate structures that have been damaged by disease. Biomaterials and techniques that intend to obtain regeneration of the periodontal apparatus in a straightforward and predictable manner have undergone considerable development over time.^15^ However, as periodontal tissues become more affected by periodontal disease, regeneration becomes an increasingly unpredictable outcome.^11^ For regeneration of intrabony defects, enamel matrix proteins are now regarded as the “gold standard” of regenerative materials after overwhelming evidence described in in-vitro,^6^^,^^25^^,^^49^^,^^58^^,^^104^ animal,^12^^,^^18^^,^^35^^,^^61^^,^^73^^,^^75^^–^^77^^,^^81^^,^^82^ and human histological studies.^9^^,^^28^^,^^50^^,^^53^^,^^54^^,^^71^^,^^83^^,^^103^ Over the last decades, Emdogain (EMD, Straumann; Basel, Switzerland) has been used for the treatment of a variety of conditions in hundreds of randomised clinical trials and over 1 million patients worldwide; no allergic reactions or adverse events have been reported.^59^^,^^78^ EMD has demonstrated statistically significant clinical attachment level (CAL) gain and pocket probing depth (PPD) reduction,^21^^,^^29^^,^^94^ radiographic defect fill,^23^^,^^74^ and higher soft tissue density^97^ compared to controls in treating intrabony defects. Most studies demonstrated that treating infrabony defects using EMD yields substantially better results when compared to OFD alone.^21^^,^^23^^,^^29^

A recent histological evaluation of the potential effects of HA on periodontal wound healing and regeneration, which was conducted in dogs with experimentally induced two-wall intrabony defects, offered novel histological evidence of bone, root cementum, and periodontal ligament formation, proposing that the observed clinical improvements may, in fact, be proof of periodontal regeneration.^87^ Despite the aforementioned properties, only a few studies on the potential of HA in periodontal intrabony vertical defect regeneration could be found in the literature.^3^^,^^7^^,^^17^^,^^41^^,^^51^^,^^96^ So far, only one clinical study compared HA with EMD in intrabony defects; however, it used a single-flap surgical technique (SFA), which is not always suitable for all types of intrabony defects.^66^

Hence, the present study aims to clinically and radiographically evaluate the adjunctive effects of cross-linked hyaluronic acid gel application when compared with enamel matrix derivates in the regenerative periodontal surgery of intrabony vertical defects.

## MATERIALS AND METHODS

### Study Design

**Fig 1:**
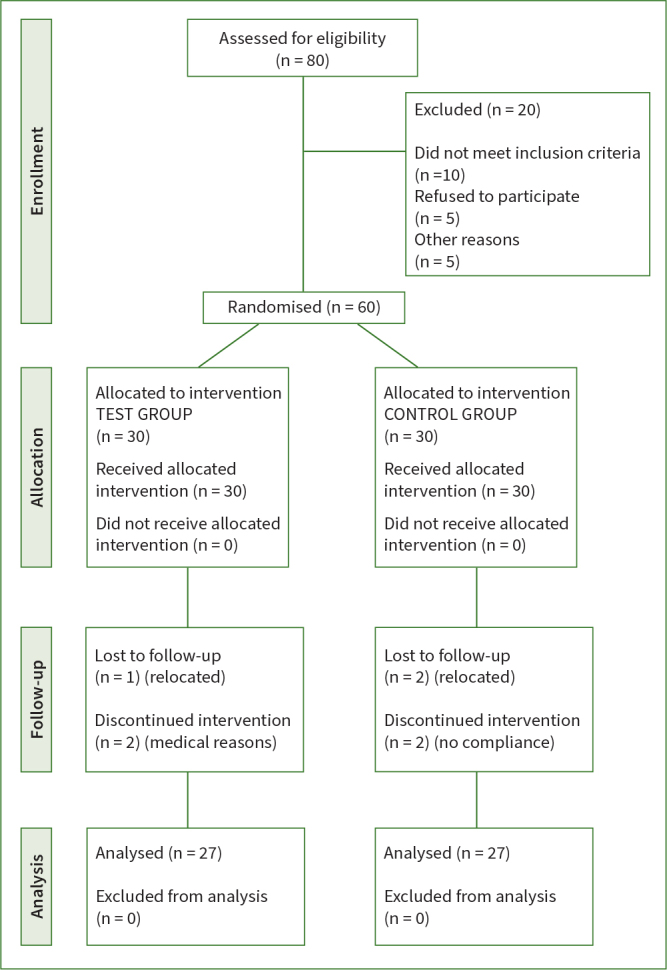
**Fig 1** CONSORT diagram.

This randomised prospective single-blind clinical study was conducted with a parallel design of two independent groups by a 1:1 allocation ratio, to evaluate the effectiveness of cross-linked hyaluronic acid when combined with regenerative surgery for intrabony defects. The test group was treated with the application of cross-linked HA gel composed of a mixture of cross-linked (1.6%) and natural (0.2%) hyaluronic acid (hyaDENT BG, Bioscience; Dümmer, Germany), while the control group was treated with EMD. In both groups, identical surgical procedures were carried out. The clinical outcomes were evaluated at baseline and six months after the procedure. The investigation was conducted in compliance with the most recent guidelines for clinical research (CONSORT guidelines) (http://www.consort-statement.org). In (Fig 1, the CONSORT diagram is displayed. The study protocol was approved by the Scientific Research Ethics Committee of the University of Medicine and Pharmacy “Victor Babes” Timisoara (Nr. Av 11/ 20.05.2019). The study was conducted between June 2019 and December 2023 and was registered in the ISRCTN Registry of Clinical Trials (ISRCTN22392064). The protocol was performed in accordance with the Good Clinical Practice (GCPs) guidelines (1996) and the Declaration of Helsinki of 1975, as revised in 2013.

### Patient Selection

Between June 2019 and June 2023, all patients receiving periodontal treatment at the Department of Periodontology, Faculty of Dentistry, University of Medicine and Pharmacy “Victor Babes” Timisoara, Romania, were screened for this study. A total of 60 nonsmoking, systemically healthy patients suffering from periodontitis stages II and III, grades A and B, and presenting each only one intrabony defect participated in this trial.^93^

The inclusion criteria were: 1) no systemic diseases that could inﬂuence the outcome of the therapy; 2) presence of 2–3 walls IBD ≥ 3 mm (distance measured radiographically between the alveolar crest and base of the defect on an intraoral periapical radiograph); 3) an interproximal PPD ≥ 6 mm; 4) 6 weeks after subgingival instrumentation (step 2 of periodontal treatment) at the experimental sites; 5) good oral hygiene – plaque index (PI<188); 6) non-smokers.

The exclusion criteria comprised: 1) patients with systemic diseases known to affect the outcome of periodontal therapy; 2) immunocompromised individuals; 3) pregnant or lactating females; 4) tobacco use in any form; 5) non-compliant patients; 7) prolonged antibiotic treatment or anti-inflammatory treatment within 6 months prior the surgery; 8) grade C periodontitis; 7) furcation involvement in the same tooth; 9) mobility grade II/III 45; 10) poor oral hygiene (PI > 1 after re-evaluation of step 2 periodontal therapy); 11) patients with parafunctional habits; 12) patients who had periodontal surgery in the last 6 months; 13) orthodontic treatment during the previous year; 14) occlusion trauma; 15) interproximal open contact points; 16) one-wall or combined one- and two-wall defects confirmed upon surgical exposure.

All study participants provided written informed consent.

### Sample Size Calculation

To estimate the sample size for our study comparing the efficacy of xHyA versus EMD on CAL improvements as the main outcome variable, we anticipated an average difference in CAL changes of 3 mm between the two treatment groups. Assuming a standard deviation of 1.5 mm, based on similar previous studies,^8^^,^^22^^,^^51^^,^^66^ a statistical significance level (alpha) of 0.05 for a two-tailed test, and aiming for a study power of 80% to detect this difference if it truly exists, we conducted a sample size calculation. Using these parameters, our calculation suggested that a total of 40 defects (20 per group) would provide the necessary statistical power to detect the anticipated difference in CAL changes between the two groups, thus minimising the risk of type II errors.

### Randomisation, Allocation, and Concealment

In order to account for possible dropouts, sixty patients and their selected sites were randomly assigned into control and test groups according to computer-generated tables (www.randomization.com) with a 1:1 allocation ratio. The control group included 30 sites treated with EMD, while the test group consisted of 30 sites treated with HA gel. For both the test and control groups, every surgical procedure was executed by a single experienced operator (O.V.), a specialist in periodontology, employing identical techniques. A blinded investigator (D.R.) was responsible for conducting all clinical and radiographic assessments.

### Clinical and Radiographic Measurements

A masked and self-calibrated investigator (D.R.) used the same periodontal probe (PCPUNC-157, Hu-Friedy; Chicago, IL, USA) to record all clinical parameters (at six points per tooth: mesio-buccal, mid-buccal, disto-buccal, mesio-oral, mid-oral, disto-oral) one week before the surgical procedure and 6 months after. The above-mentioned clinical parameters are described in the following: PPD is defined as the distance from the gingival margin to the bottom of the pocket, and gingival recession (REC), defined as the distance from the gingival margin to the cementoenamel junction (CEJ). Both were recorded to the nearest millimeter at the deepest location of the selected interproximal site. CAL is defined as the distance from the CEJ to the bottom of the pocket and calculated as the sum of PPD and REC, bleeding on probing (BOP) (Ainamo and Bay, 1975) was recorded dichotomously at the surgical site as the presence or absence of bleeding. Full-mouth plaque score (FMPS) is defined as a percentage of tooth sites revealing the presence of plaque^63^ and full-mouth bleeding score (FMBS) is defined as a percentage of tooth sites with BOP;^43^ both were recorded as the percentage of total surfaces (six aspects per tooth).

The deepest measured point was used for statistical analysis. Custom-made occlusal acrylic stents were used to standardise the probe angulation and position for the measuring at baseline and 6 months.

Soft tissue healing was assessed using the early healing index (EHI) according to the following scale: EHI 1: Complete flap closure – no fibrin line in the interproximal area; EHI 2: complete flap closure – fine fibrin line in the interproximal area; EHI 3: complete flap closure – fibrin clot in the interproximal area; EHI 4: incomplete flap closure – partial necrosis of the interproximal tissue; EHI 5: incomplete flap closure – complete necrosis of the interproximal tissue.^100^

Two (pre- and postoperative) radiographs were taken using the long-cone parallel technique for radiographic measurements. Using radiographic imaging software (CliniView Imaging Software, Instrumentarium Dental/PaloDEx Group; Tuusula, Finland), two parameters were analysed on the radiographs: defect depth (BC-BD) in mm, being the vertical distance between the bone crest (BC) and the bottom of the bone defect (BD; the site on the root surface at which the periodontium width was normal), and defect width (DW) in mm, being the horizontal distance between the root surface and bone defect margin in the most coronal part of the bone crest.

### Intra-examiner Calibration

Intra-examiner calibration was performed by measuring 30 sites deeper than 4 mm, using the same type of periodontal probe, from 5 different patients. The investigator evaluated the patients on two separate occasions, 48 h apart, before beginning the study. Calibration was accepted when measurements at baseline and after 48 h resulted in a difference of no more than 1 mm at the 95% level. The intra-examiner calibration for reliability testing resulted in κ = 0.95.

### Presurgical Therapy

Periodontal diagnosis was made according to the new classification system for periodontal and peri-implant diseases and conditions.^93^ The treatment of stage I–III periodontitis patients was conducted according to the EFP S3-level clinical practice guideline. Each individual underwent step 1 of periodontal therapy including supragingival dental biofilm control, motivation and instructions for oral hygiene instructions (OHI), adjunctive therapies for gingival inflammation, professional mechanical plaque removal (PMPR) – which includes professional interventions aimed at removing supragingival plaque and calculus – as well as possible plaque-retentive factors that impair oral hygiene practices.^79^ Step 2 of therapy, aimed at controlling the subgingival biofilm and calculus (subgingival instrumentation [SI]) was performed according to the full-mouth disinfection protocol,^69^ using ultrasonic (Acteon Group; Merignac, France), and hand instrumentation with Gracey curettes (Hu-Friedy), under local anesthesia, and was completed using air polishing (PROPHYflex 3 , KaVo KERR; Orange, CA, USA).^90^ OHI were reinforced after SI. The patients were scheduled for a follow-up appointment 6 weeks after subgingival instrumentation, which is considered the optimal time for re-evaluation.^86^ The periodontal re-evaluation was conducted to assess the achievement of therapy goals and to verify the eligibility of the defect sites for the study, if necessary. One suitable site with the deepest interproximal PPD ≥ 6 mm after step 2 of therapy was selected in each patient.

### Surgical Therapy

The surgical procedures were carried out under completely aseptic conditions. Subjects were asked to rinse with 0.20% chlorhexidine digluconate (Dentaton Intensivo, Dental Greenline, GHIMAS; Bologna, Italy) as presurgical mouthrinsing. The surgeries were performed using identical techniques for both test and control groups. After local anesthesia (4% Ubistesin Forte, 3M ESPE; Seefeld, Germany), intrasulcular incisions were performed using 12D and 15C scalpel blades (Hu Friedy), extending one tooth mesial and distal to the defect site when needed to allow access for visualisation and instrumentation of the defect. A mucoperiosteal flap was elevated on the buccal and oral aspect of the defect. Vertical releasing incisions were made only if necessary. After flap reflection, granulation tissue was removed from the defect using Gracey curettes (Hu-Friedy), and the intrabony defect was examined to confirm anatomical inclusion criteria. The infiltrated epithelium on the flap margins was also removed, and the cementum on the root surfaces was planed using hand instruments (Gracey curettes, Hu-Friedy) and ultrasonic scalers (Satelec Newtron, Acteon Group). The cleaning was perfected using an airflow prophy jet (PROPHYflex 3, KaVo KERR) and bone defects were irrigated using a saline solution. The defect was then assigned to either the test or control group by opening the sealed envelope once site preparation was completed. 

In the control group, the root surfaces were conditioned for 2 min with EDTA gel (sterile 24% EDTA gel, pH 6.7; PrefGel, Straumann) to remove the smear layer,^5^ after which any EDTA residues were removed by rinsing with a sterile saline solution. Afterward, EMD gel was applied on the root surface and into the intrabony defect.

In the test group, xHyA gel was applied directly to the defect, according to the manufacturer’s instructions, using a Uniject syringe (Hu-Friedy Anaesthetic Aspirating Syringe, 1.8cc, Type CW, 1/pk) with disposable 30-g, 0.3x12-mm needles (Sopira, Heraeus Kulzer; Hanau, Germany).

**Fig 2:**
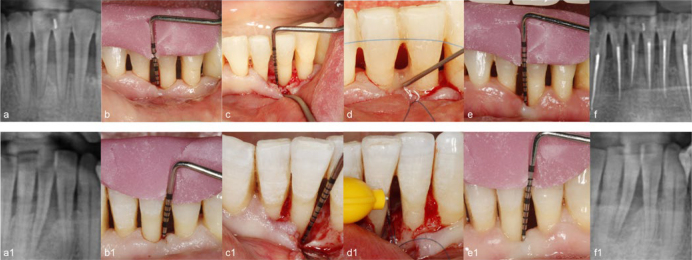
**Fig 2** Illustration of test (a–f) and control (a1–f1) group cases: a) baseline radiograph; b) baseline clinical view; c) intraoperative view of the defect; d) Hyadent (HA) application; e) 6-month follow-up clinical view; f) 6-month follow-up radiographic view; a1) baseline radiographic view; b1) baseline clinical view; c1) intraoperative view of the defect; d1) Emdogain (EMD) application; e1) 6-month follow-up clinical view; f1) 6-month follow-up radiograph.

The periosteum at the base of the flaps was carefully dissected in both treatment groups to alleviate tension and the flaps were finally repositioned at the pre-surgical or slightly coronal level without any tension and sutured using the vertical mattress suturing technique with monofilament non-resorbable 6-0 nylon suturing material (PermaSharp, Hu Friedy). Extreme care was taken to obtain primary closure of the interdental soft tissues. The sutures were removed 14 days post-operatively. Representative control and test group images from the surgical therapy phase, radiographic baseline, and 6-month follow-up are shown in (Fig 2.

### Postoperative Care

All patients were advised to rinse twice daily with a 0.20% chlorhexidine digluconate solution (Dentaton Intensivo, Dental Greenline) for 2 weeks. Mechanical tooth cleaning was not allowed in the surgical area during this period. Anti-inflammatory medication (Ibuprofen, 3 x 400 mg/day) was administered when needed. The patients attended post-operative follow-up appointments daily for the first 5 post-operative days, and sutures were removed after 14 days. Patients were recalled for weekly control up to 1 month, and then 6 months after the surgery. Post-operative care included professional plaque removal whenever necessary, and reinforcement of OHI.

### Outcome Measures 

Follow-up clinical and radiographical measurements were conducted 6 months after regenerative surgery. Statistical analysis only included measurements taken at baseline and 6 months after surgery at the deepest site of the intrabony defect. The primary outcome variable was considered CAL. Secondary outcomes were PPD, REC, BOP, EHI, BC-BD, DW FMPS, and FMBS.

### Statistical analysis

Data management and statistical analyses for this study were conducted using Microsoft Excel (version 2019, Microsoft; Redmond, WA, USA) for data collection and management, and Python (version 3.8, Python Software Foundation; Wilmington, DE, USA) for advanced data analysis and graphic representation.^99^ Continuous variables, such as CAL, PPD, REC, BC-BD and DW, were presented as mean ± standard deviation (SD). Categorical variables, including demographic characteristics, EHI, FMPS, FMBS, and BOP scores, were summarised using frequencies and percentages. Comparative analyses between the test and control groups were performed using independent-samples t-tests for continuous variables to evaluate mean differences pre- and post-treatment. The chi-squared test was applied to categorical data to examine the distribution differences between groups, or Fisher’s exact test if frequency assumptions were not met. A p-value <0.05 was considered statistically significant, indicating statistically significant differences between the treatment outcomes of the two groups. This statistical approach ensured a comprehensive evaluation of the efficacy and safety of the treatments, providing reliable and valid results. All data analysis was conducted by a biostatistician who was unaware of the group allocation.

## RESULTS

### Study Population

Sixty systemically healthy adult subjects were recruited for this study, 30 patients for each group. After the 6-month follow-up and completion of the study, data from 54 patients, 27 in each group, were available for statistics. Drop-outs from the test group were due to 1 patient relocating and 2 patients interrupting treatment for medical reasons, while in the control group, 2 patients relocated and 1 patient became non-compliant. The study population consisted of 31 females and 22 males, aged 30 to 50 years. The test group consisted of 16 females and 11 males with a mean age of 46.5 ± 7.4. The control group included 15 females and 12 males with a mean age of 43.0 ± 6.8. Analysis of the participants’ sex distribution indicated a balanced representation between men and women within both groups (40.7% women vs 44.4% men) with no statistically significant difference observed (p = 0.783). The age distribution showed no statistically significant differences (p = 0.075).

Each subject received treatment for one intrabony defect. No statistically significant intraoperative or post-operative complications were observed in any of the patients. Demographic data is described in Table 1.

**Table 1:** Demographic characteristics

Variable	Test group (n = 27)	Control group (n = 27)	p-value
Gender (female/male)	11 (m); 16 (f)	12 (m); 15 (f)	0.783^a^
Age (mean ± SD)	46.5 ± 7.4	43.0 ± 6.8	0.075^b^

SD: standard deviation; test group: HA treatment; control group: EMD treatment. ^a^Chi-squared test; ^b^Student’s t-test (unpaired). Statistical significance at p < 0.05.

At baseline, intergroup comparison between test and control treatments revealed no statistically significant differences across all measured periodontal health variables. CAL showed almost identical means (9.1 ± 2.3 mm for the test group vs 8.9 ± 2.2 mm for the control group, p = 0.822), and similar trends were observed for PPD, REC, BC-DD, and DW. These results suggest that at the onset of the study, both the test group and control group started with similar baseline conditions (Table 2).

**Table 2:** Intergroup comparison of baseline measurements

Variable	Test (n = 27)	Control (n = 27)	p-value
CAL (mm)	9.1 ± 2.3	8.9 ± 2.2	0.822
PPD (mm)	5.9 ± 2.0	5.7 ± 1.9	0.136
REC (mm)	3.1 ± 1.3	3.0 ± 1.2	0.753
BC-DD (mm)	5.3 ± 1.6	5.2 ± 1.5	0.755
DW (mm)	3.7 ± 0.9	3.7 ± 0.9	0.925

All data are expressed as means and standard deviations. BC-BD: defect depth; DW: defect width; PPD: pocket probing depth; CAL: clinical attachment level; REC: gingival recession; SD: standard deviation; test: HA treatment; control: EMD treatment. Student’s t-test, statistically significant at p < 0.05.

### Clinical and Radiographic Parameters 

The clinical and radiographic parameters are described in Table 3. At the 6-month follow-up, all measured clinical and radiographical parameters displayed a statistically significant improvement in both groups in the intragroup comparison.

**Table 3:** Clinical variables at baseline (T0) and 6 months (T1)

Parameter	Group	N	Baseline (T0)		6 months (T1)		Baseline to 6 months		p-value, intra-group	p-value, inter-group
			Mean	SD	Mean	SD	Mean	SD		
CAL	Test	27	9.07	2.34	5.89	1.83	3.18	1.49	0.001	0.132^b^
	Control	27	8.93	2.21	5.81	1.75	2.58	1.39	<0.001	
PPD	Test	27	5.85	1.95	3.85	1.16	2.00	1.46	<0.001	0.919^b^
	Control	27	5.74	1.88	3.78	1.09	1.96	1.42	<0.001	
REC	Test	27	3.07	1.34	4.11	1.22	1.04	1.29	<0.001	0.838^b^
	Control	27	2.96	1.22	4.07	1.18	1.11	1.22	<0.001	
BC-BD, mm	Test	27	5.34	1.59	2.67	0.82	2.67	1.20	<0.001	0.718^b^
	Control	27	5.22	1.48	2.57	0.78	2.65	1.08	<0.001	
DW, mm	Test	27	3.74	0.86	1.82	0.57	1.92	0.72	<0.001	0.789^b^
	Control	27	3.68	0.89	1.70	0.73	1.98	0.91	<0.001	

BC-BD: defect depth; DW: defect width; PPD: pocket probing depth; CAL: clinical attachment level; REC: gingival recesion; SD: standard deviation; test: HA treatment; control: EMD treatment. ^b^Student’s t-test. Statistically significant at p < 0.05.

**Fig 3:**
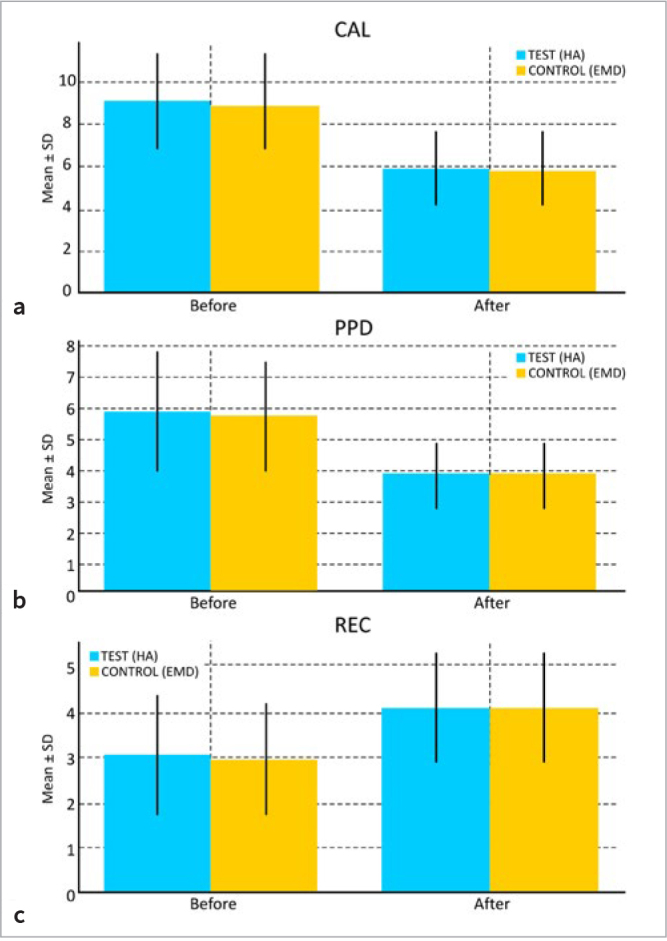
Fig 3 Means ± SD of clinical parameter changes in test (HA) and control sites (EMD) at 6 months: a) CAL gain; b) PPD reduction; c) REC increase.

- CAL: Mean CAL improved from 9.1 ± 2.3 mm to 5.9 ± 1.8 in the test group and from 8.9 ± 2.2 mm to 5.8 ± 1.8 mm in the control group, a statistically significant intragroup change (p < 0.001).
- PPD: Mean PD decreased from 5.6 ± 2.0 mm to 3.9 ± 1.2 mm in the test group and from 5.7 ± 1.9 mm to 3.8 ± 1.1 mm in the control group, a statistically significant intragroup difference (p < 0.001).
- REC: Mean REC increased from 3.1 ± 1.3 mm to 4.1 ± 1.2 mm in the test group and from 3.0 ± 1.2 mm to 4.1 ± 1.9 mm in the control group, a statistically significant intragroup difference (p < 0.001).
- Changes in mean CAL, PD, and REC are graphically described in (Fig 3.

- BC-BD, DW. Radiographically, both groups showed statistically significant improvement in BC-BD and DW. In the test group, the mean BC-BD decreased from 5.3 ± 1.6 mm to 2.7 ± 0.8 mm (p < 0.001) in the intragroup comparison, while the intergroup comparison did not change statistically significantly (p = 0.718). In the test group, the mean DW decreased from 3.7 ± 0.9 mm to 1.8 ± 0.6 mm (p < 0.001) in the intragroup comparison, while the intergroup comparison resulted in a non-statistically significant change (p = 0.789).

**Fig 4:**
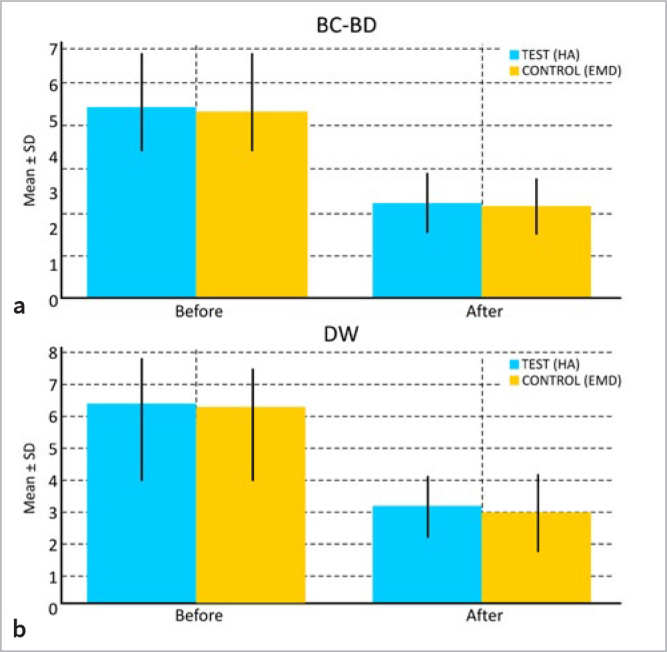
(Fig 4 Means ± SD of clinical parameter changes in test (HA) and control sites (EMD) at 6 months: a) BC-BD fill; b) DW fill.

Changes in mean BC-BD and DW are graphically described in (Fig 4.

- FMPS: Before the intervention, both the test group and the control group exhibited similar FMPS percentages, with means of 20.6 ± 1.7 and 20.5 ± 1.9, respectively. Following treatment, both groups demonstrated statistically significant improvements in plaque control, as demonstraed by FMPS decreasing to 19.2 ± 1.1 in the test group and 18.9 ± 1.5 in the control group (Table 4).

**Table 4:** Table 4 Clinical variables at baseline (T0), and 6 months (T1)

Parameter	Group	N	Baseline (T0)		6 months (T1)		Baseline-6 months		p-value intra-group	p-value inter-group
			Mean	SD	Mean	SD	Mean	SD		
EHI (%)										
1	Test		-		18 (66.67%)					0.612a
	Control		-		16 (59.26%)					
2	Test		-		7 (25.93%)					
	Control		-		10 (37.04%)					
3	Test		-		2 (7.41%)					
	Control		-		1 (3.70%)					
BOP (%)	Test		14 (51.85%)		8 (29.63%)				0.096	0.339a
	Control		18 (66.67%)		5 (18.52%)				<0.001	
FMPS %	Test	27	20.59	1.65	19.19	1.11	1.40	1.34	<0.001	0.455b
	Control	27	20.52	1.87	18.85	1.49	1.67	1.30	<0.001	
FMBS %	Test	27	18.81	1.64	17.26	1.40	1.55	1.31	<0.001	0.824b
	Control	27	18.74	1.85	17.11	1.47	1.63	1.33	<0.001	

All data are expressed as means and standard deviations. SD: standard deviation; test group: HA treatment; control group: EMD treatment; EHI: early wound healing index; BOP: bleeding on probing; FMPS: full-mouth plaque score; FMBS: full-mouth bleeding score;. ^a^Chi-squared test; ^b^Student’s t-test. Statistically significant at p < 0.05.

- FMBS: The FMBS assessments revealed statistically significant improvements in bleeding on probing, with mean scores decreasing from 18.8 ± 1.6 to 17.7 ± 1.4 in the test group and from 18.7 ± 1.9 to 17.1 ± 1.5 in the control group. These changes are described in Table 4.
- BOP: The prevalence of BOP decreased to 29.6% in the test group and 18.52% in the control group, a statistically significant change in the intragroup comparison (p < 0.001). This reduction reflects a substantial improvement in periodontal health, with 70.4% of the test group and 81.5% of the control group showing no post-treatment BOP (Table 4).
- EHI: The EHI further substantiated the efficacy of both treatments, with most sites in the test group (66.7%) and the control group (59.3%) reaching the best possible score (EHI 1), indicating complete flap closure without any complications. However, intergroup differences were not statistically significant (Table 4).

**Fig 5:**
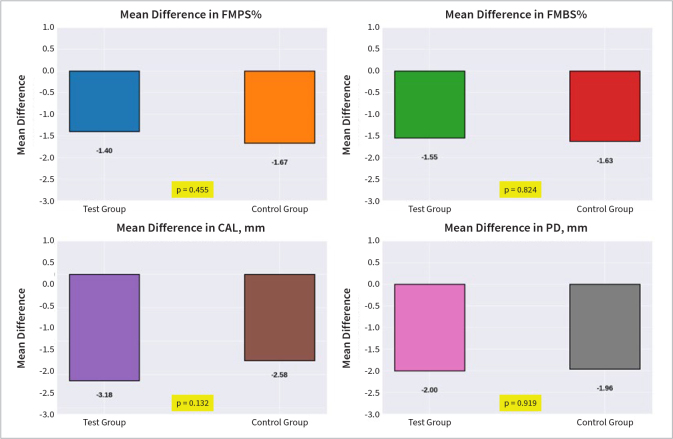
(Fig 5 Improved clinical parameters. FMPS: full-mouth plaque score; FMBS: full-mouth bleeding score; CAL: clinical attachment level; PPD: pocket probing depth.

Improved clinical changes for CAL, PPD, FMPS, and FMBS are plotted in (Fig 5.

### Frequency Distributions 

CAL gain < 3 mm was observed in almost half of the sites from the test group (48.1%) while more than half of the sites from the control group displayed a 3-4 mm CAL gain (63.0).

A reduction of 2-3 mm PPD was observed in almost half of the test group sites (44.4%), while in the control group, this reduction was seen in 25.9% of the treated sites. Almost half of the treated sites in each group experienced a PPD reduction of 4-5 mm.

**Fig 6:**
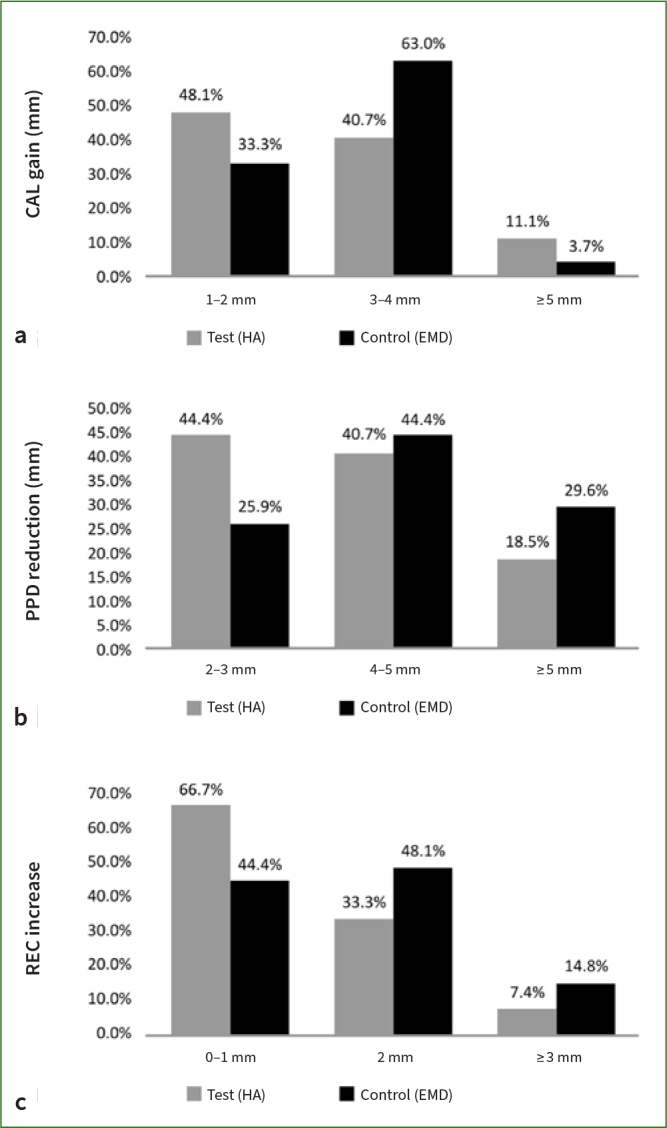
(Fig 6 Frequency distribution of clinical parameters changes (expressed as % of sites) at 6 months. a. CAL gain; b. PPD reduction; c. REC reduction.

In the test group, 66.7% of treated sites presented a small increase in REC (≤ 1 mm), while almost half of the EMD-treated sites (48.1%) in the control group showed a REC of ≥ 2 mm. Changes in the frequency distribution of CAL gain, PPD reduction, and REC increase are depicted for both groups at 6 months in (Fig 6.

The data presented in this study are available upon request from the corresponding author.

## DISCUSSION

Various studies have demonstrated that EMD promotes early wound healing and has successful outcomes in terms of improved clinical parameters. Alone or in combination with other materials and surgical techniques, EMD has become the “gold standard” when it comes to periodontal regeneration. A limitation is the need for carefully controlled conditions to obtain a successful effect. For instance, blood contamination of the root surface impairs the adsorbtion of EMD, thus having a negative impact on the regenerative outcome.^56^^,^^57^

Hence, other biomaterials that are easier to use and manipulate have been developed in the attempt to avoid EMD’s limitations. xHyA promises to overcome these deficiencies. Hygroscopic glycosaminoglycans like HA have the ability to modulate wound healing by attracting growth factors and thus influence tissue regeneration.^95^ Therefore, having direct contact with blood at the surgical site presumably does not impair the efficacy of this biomaterial. xHyA has been used in periodontics for the treatment of various conditions, such as gingivitis^37^ periodontitis,^56^^,^^57^ and more recently, gingival recessions.^26^^,^^44^^,^^67^

The present randomised trial compared the clinical results 6 months after the treatment of intrabony defects with xHyA (test group) vs the results with EMD (control group). Concerning CAL gain, although both groups demonstrated statistically significant within-group improvement, the between-group comparison of these mean differences did not reveal a statistically significant difference (p = 0.132), suggesting that the two treatments were similarly efficacious in enhancing clinical attachment levels. These results are in accordance with those observed in a recent study that also reported no statistically significant differences between groups regarding clinical measurements.^66^ However, these results are in disagreement with previous clinical trials,^22^^,^^51^ in which a statistically significant CAL gain was found in the test group, most likely because in the control group, the treatment was done using only OFD alone.

Regarding the PPD in the present study, the transition from deeper pockets (6–10 mm and >10 mm) to shallower ones (≤5 mm) was statistically significant within each group (p < 0.001), demonstrating substantial clinical improvement. Despite these marked improvements within each group, the post-treatment comparison of PPD range shifts between the test and control groups showed no statistically significant difference (p = 1) at 6 months, further supporting the conclusion that the two therapeutic approaches are comparable in terms of managing and improving PPD in patients with intrabony periodontal defects. These findings are in accordance with those of previous clinical research that obtained similar statistically non-significant results for the inter-group comparison at the 6-month mark.^22^ However, the results do not confirm those of a recent clinical trial which reported a statistically significant result in the intergroup comparison, most likely because no additional biomaterial was used for the control group.^51^ The comparison between the test and control groups shows different trends in the frequency of PPD changes. A PPD reduction of 4-5 mm was observed in both groups at similar percentages (40.7% test vs 44.4% control), thus suggesting that both treatments obtained similar results regarding PPD reduction outcome.

Gingival recession is of paramount importance for periodontal patients and is one of the undesirable consequences of surgical procedures designed to reduce residual PPD. After 6 months, the mean increase of REC in the test group was 1.0 ± 1.3 mm and 1.1 ± 1.2 mm in the control group. This increase in REC was also observed in previous studies,^22^^,^^66^ which reported greater gingival recession in the test groups vs baseline measurements. When examining the distribution of REC ranges, both groups demonstrated a trend toward an increase of REC post-treatment. Before the intervention, most of the sites in both groups had a REC of ≤5 mm. However, post-treatment, there was a noticeable shift with a decrease in the proportion of sites within this range and an increase in those with REC ranging from 6-10 mm, reflecting the clinical impact of both treatments in modifying gingival contours. A recent clinical report described similar results regarding gingival recession, with 9 out of 16 patients presenting a recession of 1 mm in the EMD group as compared to xHyA, where only 5 out of 16 patients presented the same amount of recession; those authors speculated that the probable reason is the greater contraction of the gingival tissue with adjunctive Emdogain application.^66^ The post-treatment inter-group analysis did not prove to be statistically significant (p = 1), further emphasising that REC remains a parameter still affected by periodontal surgical treatments, no matter what additional biomaterial is used. However, these findings are not in accordance with a recent study^51^ in which the intergroup analysis found greater gingival recession in the control group, with the same explanation – in the control group, infrabony defects were treated only with OFD and no additional biomaterial was used.

The present study evaluated the defect fill comparing two parameters obtained from intraoral periapical radiographs taken at baseline and follow-up. Mean values and standard deviations for BC-BD and DW are plotted in (Fig 5. Both radiographic parameters revealed a statistically significant difference between the two time points in both groups, but with no significant difference in the intergroup comparison, suggesting that both treatment courses may lead to similar percentages of defect fill. These results are in accordance with similar research that used a solid derivative of HA with OFD in 40 subjects and obtained similar results after 24 months in the intragroup comparison in the test group.^8^ Another study that used esterified HA and autologous bone to surgically correct intrabony defects also obtained similar results at 24 months in the intragroup comparison; however, there was no comparison with a control group.^3^ The comparison with these previous studies should be interpreted with caution because of the different HA formulations used, different time points at which the parameters were analysed, and most importantly, the fact that control groups were formed using only the surgical treatment technique. This is in contrast to the present study, which compared two biomaterials using the same surgical technique for infrabony defects.

The EHI was initially developed by Watchel et al^100^ and it was used to classify wound healing into five degrees that rated wound closure, fibrine presence, and necrosis presence. In our study, in most defects, an EHI of 1 was observed at the 1-week follow-up, with 66.7% in the test group and 59.3% in the control group, suggesting that both treatment procedures facilitate comparable tissue healing outcomes. These results are in accordance with very recent research, in which HA was applied into sockets after extractions, showing excellent healing of 50% in the test group.^60^ However, the results are not in accordance with a previous study from 2008, where no additional benefits of HA placed over incisions in the oral cavity were obtained.^24^ These contradictory results suggest that HA improves wound healing at a much greater level when in contact with the underlying connective tissue.

Dental biofilm was successfully managed throughout the present study in both groups, as evidenced by the reduction of FMPS, FMBS, and BOP observed at the 6-month follow-up.^36^ These results are in accordance with previous studies using HA^37^^,^^38^ and EMD.^92^ However, this can also be attributed to the recall protocol and the re-inforcement of OHI at every appointment, as well as the selection criteria that included only patients with low FMPS and FMBS.

None of the 54 patients that completed the study reported any signs of toxic response, immunogenic reactions, or other complications. This supports two previous reports that used xHyA gel and the hyaDENT BG preparation in particular for the treatment of various periodontal conditions,^66^^,^^67^, and also previous reports that used Emdogain for the same purposes.^34^^,^^84^

The present clinical study has certain limitations, the first possibly being the surgical technique employed for treating intrabony defects. Although the OFD technique is used in a wide variety of clinical trials,^22^^,^^51^^,^^68^ certain flap designs have been suggested in order to achieve primary closure, preserve interdental tissues, limit flap elevation, improve wound stability, and reduce morbidities, e.g., modified papilla preservation flap (M-PPT) or simplified papilla preservation flap (SPPT).^13^^,^^14^^,^^89^^,^^102^

The second limitation could be the lack of a histological analysis which might have shed light on the type of attachment formed after the wound healing. However, due to the associated morbidity of the periodontal tissues, histological analysis was not performed in the current clinical trial. For this particular type of investigation, animal trials are better suited to reveal whether xHyA induces regeneration or repair of the periodontal tissues.

A third possible limitation is the fact that this study was limited to only one research center. Hence, although both sexes and a wide range of ages were included in both groups, a multicenter study could provide a more generalised view of the improvements additional xHyA could offer in the treatment of intrabony defects.

Further research should also focus on comparing various hyaluronic acid products for treating periodontal intrabony defects, thus offering more homogenous results. Treatment protocols for other periodontal defects, such as furcation involvement, should also explore the additional benefit of hyaluronic acid products. Furthermore, combinations of enamel matrix derivative and hyaluronic acid could also be a direction for future research. As far as we know, except for a single in-vitro investigation on the inhibition of the lipopolysaccharide-induced inflammatory response on human epithelial and bone cells by the combination of enamel matrix derivative and hyaluronic acid, this combination has not been clinically tested.^70^

In summary, the present clinical research described improvements in all clinical and radiographical measured parameters, suggesting that the additional application of xHyA in the surgical treatment of intrabony defects yields wound healing results similar to those obtained with the additional application of EMD in the same treatment.

## CONCLUSION

The findings show that both therapies produced similar statistically significant clinical and radiographical improvements after six months when compared with baseline, early wound healing included. Hence, in conjunction with a surgical approach, cross-linked hyaluronic acid appears to potentially serve as a viable substitute for enamel matrix proteins in the treatment of intrabony periodontal defects, especially when it comes to ease of surgical use. Additional clinical investigations involving a greater sample size are required to validate the current clinical and radiographical observations. Furthermore, histological studies are also warranted to assess the nature of the healing achieved.
